# Ameliorative effect of melatonin against contrast media induced renal tubular cell injury

**Published:** 2014

**Authors:** Hamid Nasri, Maryam Tavakoli, Ali Ahmadi, Azar Baradaran, Mehdi Nematbakhsh, Mahmoud Rafieian-Kopaei

**Affiliations:** 1 Hamid Nasri, Department of Nephrology, Division of Nephropathology, Isfahan University of Medical Sciences, Isfahan, Iran.; 2 Maryam Tavakoli, Department of Internal Medicine, Isfahan University of Medical Sciences, Isfahan, Iran.; 3 Ali Ahmadi, Department of Epidemiology, Shahid Beheshti University of Medical Sciences, Tehran, Iran; 4 Azar Baradaran, Department of Pathology, Isfahan University of Medical Sciences, Isfahan, Iran.; 5 Mehdi Nematbakhsh, Water and Electrolytes, Research Center, Department of Physiology, Isfahan University of Medical Sciences, Isfahan, Iran.; 6 Mahmoud Rafieian-Kopaei, Medical Plants Research Center, Shahrekord University of Medical Sciences, Sharekord, Iran.

**Keywords:** Contrast-induced nephropathy, Contrast media, Iodixanol, Melatonin, Reactive oxygen species, Renal failure

## Abstract

***Background and Objective:*** Reactive oxygen species (ROS) is a mediator of renal damage. Melatonin is a potent-free radical scavenger. Our objective was to test whether melatonin would protect against the nephrotoxicity of contrast media.

***Methods:*** In an experimental study 40 adult male Wistar rats were randomly divided into four equal groups including: 1) Control group (No drug), 2) Contrast media group (10 ml/kg iodixanol i.v. single dose), 3) Contrast media and melatonin (first 10 ml/kg iodixanol then 10 ml/kg/day melatonin by i.p. injection on days 3, 4 and 5) and 4) Contrast media and melatonin pretreatment group (melatonin 10 ml/ kg/day by i.p. injection on 1, 2 and 3 days, then 10 ml/kg iodixanol by i.v. injection on third day. The blood creatinine and BUN as well as the histological changes were evaluated for severity of renal injury (degeneration, vacuolization of tubular renal cells, dilatation of tubular lumen and presence of debris in the lumens), by scoring from one to four.

***Results:*** Contrast media significantly increased the creatinine and BUN and renal injury (p<0.05). Melatonin prevented and reversed the injury induced by contrast media (P<0.05). Pretreatment with melatonin reduced the renal injury induced by contrast media (P<0.05).

***Conclusion:*** Melatonin is an effective drug to prevent contrast–induced renal injury. Therefore its usage (especially pretreatment) might be beneficial in patients who are planning to use contrast media agents.

## INTRODUCTION

Acute renal injury after exposure to iodinated contrast media increases morbidity, hospital stay and early mortality.^1^ Contrast-induced acute renal damage is an important clinical event with an international increasing number of cases.^2^^,^^3^ Contrast-induced nephropathy (CIN) occurs in 2% to 25% of patients undergoing coronary intervention.^2^^,^^3^ CIN is defined as increase in serum creatinine of 0.5 mg/dL, or a 25% relative increase in creatinine at 48 hours after contrast exposure.^1^^-^^4^ The pathophysiology of CIN is a complex interplay between vascular and tubular effects. Tubular toxicity, generation of reactive oxygen species and medullary blood flow reduction are suggested as being the risk factors of CIN.^1^^-^^4^ After the infusion of contrast agents, osmotic load, hypoxemia of the renal medulla, viscosity and renal free radical production via post-ischemic oxidative stress will increase.^2^^-^^4^ While reactive oxygen species (ROS) have been increasingly found as the mediator of renal damage CIN, however renal medullary hypoxia is indisputable to the pathophysiology of this disease.^2^^-^^5^ The outer medulla is especially susceptible to hypoxia; oxygen necessities are high due to salt reabsorption in Henle's thick ascending limbs, while oxygen supply is scant.^4^^,^^5^ Contrast agents in the medulla affect the delicate balance between oxygen delivery and oxygen consumption by several mechanisms, the main mechanism being reduced blood perfusion. In both the cortex and the medulla, contrast agents can shift the balance between vasodilatory and vasoconstrictive substances towards vasoconstriction.^3^^-^^5^

Melatonin (*N*-acetyl-5-methoxytryptamine) is a secretory product of the pineal gland. It acts through melatonin receptors, to do various physiological processes including circadian entrainment, seasonal reproduction, retinal physiology, ovarian physiology, immune function, blood pressure regulation and oncogenesis.^6^ Furthermore, melatonin was attested to be a potent free radical scavenger and a broad-spectrum antioxidant.^6^^,^^7^ Melatonin is broadly available, relatively free of side effects, rapidly active after oral taking, and commonly used in humans in the treatment of insomnia. The melatonin function upon the kidney has been demonstrated by various experimental and human researches.^6^^,^^7^ Melatonin seems to act through multiple pathways: as an apoptosis modulator, as an antioxidant, and as a circadian modulator of vascular function. Melatonin is capable of abolishing the renal toxicity induced by some medications that may cause oxidative stress in kidney cells, including cisplatin, gentamicin, vancomycin and amikacin.^6^^,^^7^ Considering the safety profile of melatonin and its ability to reduce renal injury due to some other nephrotoxic drugs, this investigation was designed to test whether melatonin would protect against the nephrotoxicity induced by contrast media.

## METHODS

***Design and Setting:*** In this study, we conducted an experimental design in laboratory setting of the Shahrekord University of Medical Sciences to identify the effect of melatonin against the nephrotoxicity of contrast media. The present study was conducted in time period of 9 months in 2012 and included 40 adult male Wistar rats (six weeks old) with a mean body weight of 200-250g. Estimated required sample size for one- sample comparison of mean to hypothesized value with alpha 0.05 (Two-sided), power 0.95, mean and standard deviation 25(4) was 9 samples in each group. To elevate precision, we selected 10 samples in each groups.

The rats were designated randomly into four equal groups as follow:

**Group I:** control group (sham group); they did not receive any drugs.

**Group II**: contrast media group; they received iodixanol 10 ml/kg/single dose by i.v. injection.

**Group III**: contrast media and melatonin; rats in this group, first received iodixanol 10 ml/kg/single dose by i.v. injection, then they were treated with melatonin 10 ml/kg/day by i.p. injection on days 3, 4 and 5.

**Group IV**: melatonin and contrast media group; rats in this group were pretreated with melatonin 10 ml/kg/day by i.p. injection on days 1, 2 and 3, then they received iodixanol 10 ml/kg by i.v. injection.

***Experimental study protocol: ***The experiment protocol was approved by Ethical Committee of Shahrekord University of Medical Sciences (Ethical Code No: 91/11/1). All rats were given unlimited access to standard rat chow and water. On the first day, blood samples (1ml) were collected from the lateral tail vein for examination of creatinine (Cr) and blood urea nitrogen (BUN) levels. After 20 minurtes, rats of group IV were given 10 ml/kg of melatonin by daily i.p. injection for 3 days. On the third day, 10 ml/kg contrast media was injected to animals in the groups II and IV via the lateral tail vein. After 20 minutes rats of group III were given 10 mg/kg of melatonin by i.p. injection daily for 3 days. On the fifth day, all rats were anesthetized and the blood samples were collected for evaluation of Cr and BUN levels and then all rats were killed using ketamin.

The kidneys were dissected out immediately after sacrificing and fixing with 10% formalin for histological examinations. The kidney paraffin sections (2-3 µm-thick) were prepared by a microtome and stained with hematoxylin and Eosin (H&E) for histological examinations. H&E stained sections were evaluated by light microscope for severity of renal injury (degeneration, vacuolization of tubular renal cells, dilatation of tubular lumen and presence of debris in the lumens), using conventional protocol.^8^^,^^9^ The slides were coded and evaluated by a nephropathologist who was blinded to the animal groups. Each morphologic lesion was scored from one to four, while the score zero was assigned to the normal tissue without any pathological damage. Therefore, score zero was considered as normal, score one: 0-19% involvement, score two: injury 20-49%, score three; 50-69% and score four; 70-100% damage.^8^^,^^9^

***Data analysis: ***All parameters were summarized with mean and standard deviation. One-way Analysis of Variance (ANOVA) and post hoc tests (Bonferroni Test) were used for the comparison of Mean values between groups. P values of less than 0.05 were assumed to be significant (P<0.05). To calculate sample size and data analysis stata software was used.

## RESULTS

There was no difference in creatinine or BUN level between groups at the start of the experiment (Table-I). The changes in these parameters are shown in Table-II. Creatinine levels were not different between group I and group IV. However, creatinine levels were significantly higher in the group II compared to other groups (1.5 mg/dl. range 0.5 – 1.5, P=0.008). Although creatinine levels were higher in groups III and IV than in the control group, they were significantly lower than the level of group II. Creatinine level of the group III was higher than the group IV. BUN level was higher in the group II than in the other groups.

Renal histological changes including degeneration, dilatation, debris and vacuolization were higher in the group II than in other groups. Renal injury scores in groups III and IV were higher than the ones in group I but lower than the score of the group II. The comparison between groups are shown in Table III.

**Table-I:** Mean ±SD of creatinine and BUN of each group at the Start of the study

*Groups*	*BUN*		*Creatinine*	
	*Mean*	*Std. Dev.*	*Mean*	*Std. Dev.*
Control I	25.37	5	0.58	0.12
Contrast II	24.75	5	0.68	0.3
Contrast+ Melatonin III	25.37	4	0.68	0.3
Melatonin, then Contrast IV	25.37	5	0.67	0.31
Total	25.21	4.6	0.65	0.26
F (ANOVA)	0.03		0.25	
P Value	0.99		0.075	

**Table-II:** Mean ±SD of BUN, creatinine and score levels of each group at the end of the study

*Groups*	*BUN*		*Creatinine*		*Score*	
	*Mean*	*Std. Dev.*	*Mean*	*Std. Dev.*	*Mean*	*Std. Dev.*
Control I	25.62	4.9	0.58	0.11	3.12	1.4
Contrast II	26.25	1.9	1.58	0.12	48.12	4.7
Contrast+ Melatonin III	25.62	6	0.81	0.14	40.5	4.8
Melatonin, then Contrast IV	19.37	3.1	0.65	0.11	43.37	22.4
	24.218	4.9	0.90	0.42	33.78	21.3

**Table-III:** Comparison of BUN, creatinine and score levels between groups at the end of study

*Between Groups comparison*	*BUN*	*Creatinine*	*Score*
I v.s II	0.62 P>0.05	1 P=0.001*	45 P=0.001*
I v.s III	0 P>0.05	0.23 P=0.008*	37.4 P=0.001*
I v.s IV	6.3 P= 0.04*	0.07 P=0.06	40.3 P=0.001*
II v.s III	0.62 P>0.05	0.78 P=0.001*	7.6 P>0.05
II v.s IV	6.8 P= 0.02*	0.93 P=0.001*	4.7 P>0.05
III v.s IV	6.2 P= 0.04*	0.16 P=0.09	2.8 P>0.05

* P values of less than 0.05 were assumed to be significant (P<0.05).

## DISCUSSION

In this study we found the ameliorative effect of melatonin against CIN, through improvement of pathology damage scores in groups III and IV against group II. Serum creatinine level also supported the histopatologic findings. One of the interesting findings of this study was the lower serum creatinine of group IV in comparison to group III which supports the hypothesis that pretreatment with melatonin can abolish the kidney injury induced by contrast media. Kilic et al., investigated the underlying mechanism of the protective effect of melatonin against cisplatin-induced renal damage in a rat renal-toxicity model in vivo. They found that both serum creatinine and urea nitrogen decreased significantly with melatonin co-treatment with cisplatin. They also demonstrated an improvement in histological lesions of cisplatin renal damage by melatonin co-treatment.^10^ Ozguner et al. found that melatonin was capable of preventing renal tubular injury by reducing oxidative stress and protecting the kidney from oxidative damage induced by 900 MHz mobile phone.^11^ Likewise, in the study conducted by Zararsiz et al. the protective effect of melatonin against formaldehyde-induced renal damage in rats was investigated. In light microscopic evaluation of the renal tissue, they observed that melatonin was able to prevent formaldehyde-induced oxidative renal damage in rats.^12^ Recently, Lee et al. investigated the ameliorative effect of melatonin against gentamicin-induced renal toxicity and oxidative stress in rats.^13^ They also tested the effects of melatonin on induction of apoptotic cell death and its potential mechanisms in kidney tissue in response to gentamicin treatment. They observed that melatonin was able to protect kidney tissue against the oxidative damage and the nephrotoxic effect caused by gentamicin treatment.^13^ Moreover, histopathological evaluation confirmed the kidney protective effect of melatonin. They concluded that melatonin was capable of preventing renal toxicity induced by gentamicin through its potent antioxidant capability.^13^ CIN is one of the most common reasons of acute kidney injury in hospitalized patients.^14^ It increases long-term morbidity and mortality and extend the hospital stay.^14^ No effective treatment exists for this iatrogenic disease; thus, prevention remains the key strategy.^14^ Studies have also shown that infusion of contrast agents increases osmotic load, viscosity, renal free radical production through post-ischemic oxidative stress and hypoxemia of the renal medulla.^14^ However, the exact mechanism of CIN is not fully clarified. Contrast agents have direct toxic effects on renal tubular cells and renal hemodynamics, directing to selective diminution of outer medullary blood flow.^1^^-^^4^^,^^14^ Various studies, have shown that oxygen radicals play a major causative role as the primary physiological insult.^15^^-^^19^ Infusion of contrast materials with the resultant increase in osmotic load and viscosity, increases hypoxemia of the renal medulla and renal free radical production through post-ischemic oxidative stress.^14^ This is due to decreased tissue oxygen tension which promotes mitochondrial generation of ROS.^14^ ROS have been increasingly accused as the mediators of injury in ischemic, toxic, and immune-mediated tissue damages. Melatonin activates several antioxidant enzymes, modulates gene expression for several protective enzymes and reduces lipid peroxidation.^19^^,^^20^

Free radicals are chemical constituents that have an unpaired electron.^21^^-^^24^ When an electron is added to O2 then the superoxide anion radical “.O2-” is formed. “.O2-” is reduced by superoxide dismutase to H2O2 which is toxic at high concentrations and can be reduced to “.OH”. The hydroxyl radical (.OH) damages cells.^22^^-^^24^ Melatonin efficiently neutralizes the hydroxyl radical (.OH).^25^ Moreover, melatonin has been found to be effective in defending against severe free radical-mediated toxicity in a variety of conditions including: ischemia-reperfusion injury, acute kidney injury, chemotherapy, nitrogen mustard toxicity caused by mercuric chloride and gentamycin.^6^^,^^7^^,^^25^ In our study, melatonin was also capable of attenuating the CIN, which is in agreement with some other studies which have used other nephrotoxic agents.^11^^-^^13^ In fact melatonin is a safe drug^6^^,^^7^^,^^25^ and we recommend its use in high risk patients scheduled to apply contrast agent.

## CONCLUSION

Melatonin is an effective drug to attenuate contrast–induced renal injury.

## Authors’ contribution:

All authors contributed in design of the research. AH analyzed the data. HN and AB wrote the manuscript. MRK and MN edited the paper. All authors read and approved the paper.

## Notes

***Funding/Support: ***This study was conducted through university inter-relationship. The manuscript is based on a residency thesis in Isfahan University of Medical Sciences (Water and Electrolyte Research Center; proposal number #391387) and grant by Shahrekord University of Medical Sciences (grant number: 1395). The study was conducted in Medical Plants Research Center of Shahrekord University of Medical Sciences.
